# Grating-Coupled Surface Plasmon Resonance (GC-SPR) Optimization for Phase-Interrogation Biosensing in a Microfluidic Chamber

**DOI:** 10.3390/s18051621

**Published:** 2018-05-18

**Authors:** Stefano Rossi, Enrico Gazzola, Pietro Capaldo, Giulia Borile, Filippo Romanato

**Affiliations:** 1 Department of Physics and Astronomy “G. Galilei”, University of Padua, Via Marzolo 8, 35131 Padua, Italy; stefano.rossi.16@studenti.unipd.it (S.R.); enrico.gazzola@unipd.it (E.G.); filippo.romanato@unipd.it (F.R.); 2 Laboratory for Nanofabrication of Nanodevices, Corso Stati Uniti 4, 35127 Padua, Italy; 3 Fondazione Institute of Pediatric Research Città della Speranza, Corso Stati Uniti 4, 35127 Padua, Italy; 4 CNR-INFM TASC IOM National Laboratory, Area Science Park S.S. 14 km 163.5, 34012 Trieste, Italy; pietrocapaldo@gmail.com

**Keywords:** surface plasmon resonance, biosensing, nanofabrication, lab-on-a-chip, microfluidic

## Abstract

Surface Plasmon Resonance (SPR)-based sensors have the advantage of being label-free, enzyme-free and real-time. However, their spreading in multidisciplinary research is still mostly limited to prism-coupled devices. Plasmonic gratings, combined with a simple and cost-effective instrumentation, have been poorly developed compared to prism-coupled system mainly due to their lower sensitivity. Here we describe the optimization and signal enhancement of a sensing platform based on phase-interrogation method, which entails the exploitation of a nanostructured sensor. This technique is particularly suitable for integration of the plasmonic sensor in a lab-on-a-chip platform and can be used in a microfluidic chamber to ease the sensing procedures and limit the injected volume. The careful optimization of most suitable experimental parameters by numerical simulations leads to a 30–50% enhancement of SPR response, opening new possibilities for applications in the biomedical research field while maintaining the ease and versatility of the configuration.

## 1. Introduction

Biosensing, the detection of an analyte through the interaction between a biological ligand and a physical transducer, is a primary target of multidisciplinary scientific and technological research [1,2,3,4]. With advances in photonics, a large variety of optical methods have been applied to biosensing including spectroscopy, microscopy and plasmonic resonance. The development of microscopic sensing platforms in combination with microfluidic technologies has opened up the possibility of integrating sensors and arrays in lab-on-a-chip devices [5,6,7].

A well-established approach to optical biosensing is that based on surface plasmon resonance (SPR), the resonant excitation of electromagnetic modes called Surface Plasmon Polaritons (SPP), supported by metal–dielectric interfaces and consisting of electromagnetic waves coupled to conduction electrons collective oscillations [8,9,10,11]. SPR-based sensors are widely appreciated because they allow for label-free, real-time detection and can be implemented in cost-effectively integrated devices [12,13,14,15].

The first approach to provide coupling between incident light and SPP for SPR biosensing is by a prism; this is still the most widely used method in commercial SPR devices due to its high sensitivity and ease of use [16,17,18,19]. However, the cumbersome and complicated optical system of prism-based devices limits their versatility and integration capabilities in miniaturized arrays and lab-on-a-chip platforms. The rival approach is grating-based coupling (GC-SPR), in which the resonance conditions are provided by diffraction of the incident light; however, this configuration is less often adopted due to its lower sensitivity [20,21,22]. On the other hand, grating-based devices offer much higher miniaturization and integration capabilities, resulting in growing interest in this kind of sensor for lab-on-a-chip applications [20,23,24].

It is thus essential to find strategies to enhance the sensitivity of these sensors. It was demonstrated that a sensitivity enhancement can be achieved by working in “conical mounting,” a configuration where the scattering plane is rotated of an azimuthal angle with respect to the grating wave vector [25,26,27,28]. It was also observed that the symmetry breaking, due to the azimuthal rotation, involves a fundamental role of the incident light polarization on SPP excitation [25,29]. Therefore, in addition to the usual angular and wavelength interrogation techniques, the unique role of polarization in grating-coupled SPR devices can be exploited to perform a polarization scan. In this configuration, the device reflectance is a function of the incident light polarization and variations of the phase or amplitude of the resulting spectrum constitute the sensor response [30]. This method allows high resolution as well as a compact, simple and cost-effective detection setup, opening up remarkable possibilities for further integration in lab-on-a-chip devices, as already demonstrated [31].

This microfluidic plasmonic chip is a starting point that requires SPR response enhancement prior to biomedical applications. Here, we will investigate the grating coupler and working conditions with the aim to optimize the SPR response in the case of polarization scan-based biosensing in an aqueous environment.

## 2. Materials and Methods

### 2.1. Numerical Simulations

Numerical simulations were performed by Chandezon method (C-method), which is known as one of the most efficient and stable algorithms developed to compute the optical response of periodically patterned multilayer structures to an impinging monochromatic light beam [32,33]. The method has been extended and improved over the years and proved to be reliable in producing realistic reflectance spectra in cases including conical mounting [34,35], multilayer gratings of arbitrary profile [36,37] and also digital gratings [38]. The gratings were assumed to have a duty cycle of 50% and the gold film was considered as bulk, since the transmitted component was found to be negligible. The refraction index of the gold film was determined via spectroscopic ellipsometry.

### 2.2. Grating and Microfluidic System Fabrication for GC-SPR

Grating fabrication was performed following a proven procedure already described by our group, with minor adjustments [39]. In detail, digital gold gratings were produced, with a period of 400 nm and a duty cycle of 50%, on a 2.5 × 2.5 cm^2^ clean microscope slide by a Laser Interference Lithography (LIL) process. The high-temperature cleaning procedure has been performed, whereby sequential oxidative desorption and complexing with H_2_O_2_:NH_4_OH:H_2_O (1:1:3) has been implemented. A 5-nm Ti adhesion layer was first deposited via electron beam evaporation, followed by a 40-nm Au film. A polymeric stencil made of Poly(methyl methacrylate) (PMMA), with five 2 × 2 mm^2^ areas, was used during the evaporation step to divide a single chip into different areas and allow the bonding of the polydimethylsiloxane (PDMS; Sylgard^®^ 184 Silicone Elastomer, Dow Corning, Midland, MI, USA) microfluidic system directly on glass. A positive photoresist Microposit S1805 (MicroChem Corp., Westborough, MA, USA) diluted into propylene glycol monomethyl ether acetate (PGMEA; Microresist Technology GmbH, Berlin, Germany) (2:3) was spun at 4000 rpm for 30 s on an adhesion promoter (HDMS; MicroChem Corp., Westborough, MA, USA) at 2000 rpm for 30 s. LIL in lab-made Lloyd’s configuration setup was used to impress the grating’s grooves into the photoresist. The optimal dose for the required duty cycle was found to be 65 mJ/cm^2^, corresponding to exposure times around 10 min using a 325 nm He:Cd laser (Kimmon Koha Co., Tokyo, Japan), spatially filtered with a 10 μm diameter pinhole. The incidence angle was set to obtain the desired period. The resist was developed in Microposit^®^ MF 321 (Shipley Comp., Marlborough, MA, USA) for 40 s and then rinsed for 60 s in Milli-Q water (MilliPore, Burlington, MA, USA). A second 40 nm thick gold layer was deposited onto the nanostructured pattern after O_2_ plasma cleaning to remove any residual layer, aligning the stencils to the gold squares. A final lift-off process in a 1:4 solution of isopropyl alcohol (IPA) and acetone at 70 °C for 15 min allowed us to obtain all-metal gratings. The gratings were then characterized by Scanning Electron Microscopy (SEM), resulting in a period of 396±4 nm and a duty cycle of 46±1%.

**Figure 1:**
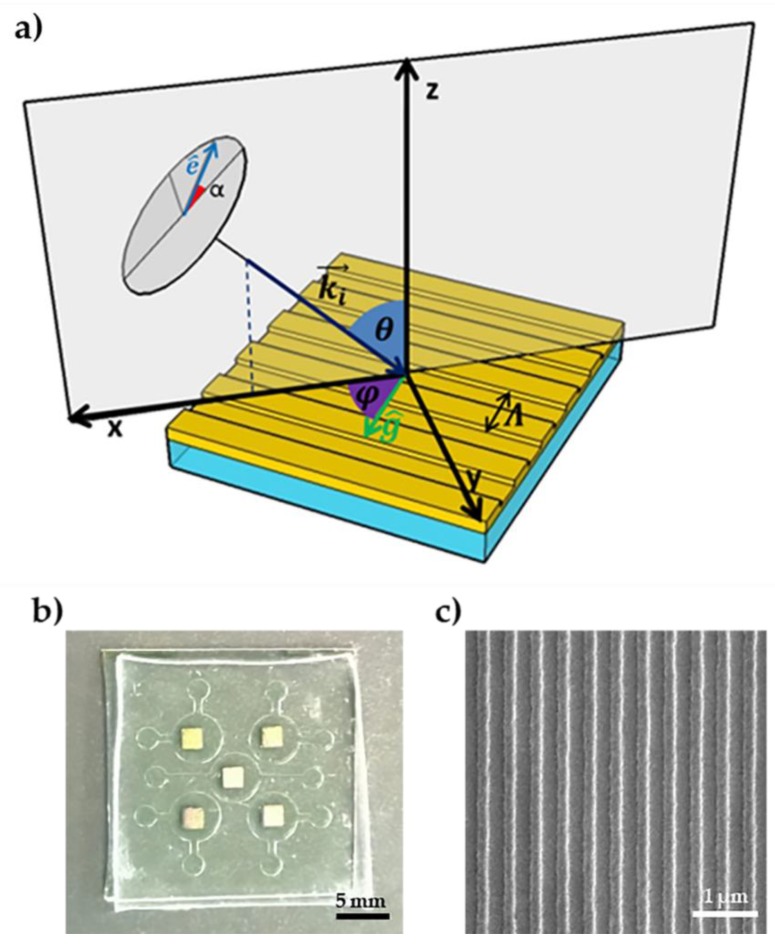
Conical mounting scheme and sensing unit. (**a**) Schematic representation of the grating-coupled SPR system based on phase interrogation. The *x* and *z* axes lay on the scattering plane. The grating wave vector $\hat{g}$ forms an angle φ with the *x*-axis, defined as azimuth, while the incident light wave vector $\vec{k_{i}}$ is incident at an angle θ from the normal to the grating surface, defined as the polar angle. The polarization α is defined as the angle formed by the electric field versor $\hat{e}$ from the scattering plane. Λ indicates the period of the grating. (**b**) Final chip integrated in the microfluidic chamber. Each glass slide is divided into five sensing areas with independent chambers. (**c**) Representative SEM image of a gold grating prior to microfluidic chamber bonding.

Microfluidic chambers were realized in PDMS with soft lithography and covalently bonded on the SPR chip with O_2_ plasma treatment and an argon plasma pre-cleaning of the gold surface, as previously described [39]. The master was pattern-transferred in a silicon substrate using a negative photoresist UV-lithographed mask via Bosh dry etching, obtaining a depth of 56 μm. The silicon master was then replicated with PDMS to obtain the desired positive master. The surface was then silanized to avoid sticking of the PDMS during replica. A graphic scheme and representative picture of the grating chip are shown in Figure 1.

### 2.3. GC-SPR Detection Setup

For SPR generation and detection, we used a custom-made bench setup based on phase interrogation, previously adopted for other applications [30,31]. Briefly, an incident collimated laser beam at 633 nm crosses a half-wave plate mounted on a motorized rotation system, before reaching the grating. The sample is mounted on a rotation stage to select the azimuth, while both the camera and laser were mounted in an articulated base to allow polar angle rotation. The reference azimuth corresponds to the orientation that minimizes the intensity of reflected light, i.e., resonance. This angle is selected rotating the grating until the minimization condition is reached and finely tuned with polarization scan around the identified position for more precise selection of the orientation.

The reflected light is then collected by a CMOS camera, whose exposure time is regulated in order to avoid signal saturation. Real-time measurements were performed monitoring averaged reflected light intensity in a selected Region of Interest (ROI) of the sensing region as a function of polarization angle. The ROI was selected in order to exclude margins were fabrication misalignments could generate unwanted reflection. The entire system was controlled by custom-made software. Experiments were conducted acquiring at least 10 points around maximal intensity sampling every 5°. For each angular position 30 frames were averaged (camera working at 30 fps).

### 2.4. Sensing and Functionalization Experiments

The sensor performance was analysed using a model sodium chloride (NaCl, SIGMA-Aldrich, St. Louis, MO, USA) solution in Milli-Q water, as in a previously adopted method to evaluate SPR response to bulk refractive index variation [39]. The considered condition was 200 mM NaCl, similar to buffers used in biosensing (e.g., PBS or HBSS).

A standard protein–vitamin interaction was used as a starting point for biosensing applications [31]. A 2 mM aqueous solution of Biotin-PEG2kDa-SH (Nanocs Inc., New York, NY, USA) was fluxed in the microfluidic chamber and SPR signal was monitored in static (stop-flow) conditions at room temperature (22°). Rapid and continuous acquisition of the SPR spectra was recorded for the first 2 h of the functionalization, then additional measurements were performed after at least 8–10 h when the functionalization was complete. The presented experiments were performed dissolving reagents in Milli-Q water (MilliPore, Burlington, MA, USA) to ensure repeatability of the analysis. Avidin (Sigma-Aldrich) was dissolved in Milli-Q water at 4 µg/mL. Avidin biosensing was monitored for at least 1 h even though the steady state was observed after 20 min.

### 2.5. Data Acquisition and Analysis

The reflected light is acquired as a function of polarization angle and fitted by the harmonic curve described below. The shift $\Delta \alpha_{0}$ is proportional to the refractive index change Δn and hence used as SPR response of our biosensing system. The sensitivity of the system is defined as the ratio between the phase shift and the refraction index variation ($S=\Delta \alpha_{0}/\Delta n$). All reported data are expressed as the mean obtained from different experimental replicas, with the corresponding standard error of the mean (s.e.m.). Salt concentrations and SPR response were compared to numerical simulations. The kinetics of binding is well approximated by the Hill function using the orthogonal distance regression with Origin^®^ (OriginLab Corp., Northampton, MA, USA). Comparison between the experimental groups was made using non-paired Student’s *t*-test, considering *p* < 0.05 statistically significant.

## 3. Results

### 3.1. Theory of Phase-Interrogation for GC-SPR in Conical Mounting

In conical mounting (Figure 1a), the reflectance was found to exhibit sinusoidal behaviour as a function of light polarization [30], which can be fitted with the formula

(1) $$R=f_{0}-f_{1}\cos\left(2\alpha +\alpha_{0}\right),$$

where *α* is the polarization, $\alpha_{0}$ the phase parameter and $f_{0}$ and $f_{1}$ control respectively the offset and amplitude of the polarization spectrum. The reflectance is minimized for $\alpha_{min}=-\alpha_{0}/2$. The phenomenon can be understood assuming that at resonance only the electric field component lying on the grating’s symmetry plane is effective for SPP excitation [29], because the perpendicular component is not diffracted and therefore no coupling is possible. That assumption brought to the analytical determination of the polarization that minimizes the reflectance at resonance, which was validated experimentally [25]. However, this model is valid only at resonance, because it assumes that all the electric field component lying on the (G, n) plane is absorbed. When going out of resonance due to a refractive index change, part of that field is reflected due to momentum mismatch between the diffracted and plasmonic wave vectors.

Out of resonance, the global reflectance can be decomposed into the three reflected components:

(2) $$R=\zeta {\left|E_{⊥}\right|}^{2}+\mu {\left|E_{∥}\right|}^{2}+\xi {\left|E_{z}\right|}^{2},$$

where $E_{⊥}$ is the component of the incident electric field that is perpendicular to the grating wavevector and lays in the *x*, *y* plane of Figure 1; $E_{∥}$ is the parallel component laying in the same plane, while $E_{z}$ is the component perpendicular to the grating surface, laying in the *z*-axis of Figure 1. The three coefficients (ζ,μ,ξ) give the fraction of the correspondent reflected incident field. Each unreflected fraction goes into plasmonic excitation and intrinsic losses in the metal. At resonance, the reflectance is determined only by the non-coupled perpendicular component, while a variation of the refraction index determines the activation of the other two components. This causes a change of the overall phase at the same azimuthal and polar angles. In general, all three dampenings are expected to have refraction index dependence. The working principle of the phase-interrogation is to monitor the fitted phase parameter change.

### 3.2. Simulation Study for Grating Line Depth Contribution to SPR Response in Phase Interrogation

The line depth (indicated as A) of the grating is a parameter that controls the plasmonic efficiency and has an effect on the global sensitivity. For prism couplers, the reflectance around resonance can be approximated by a Lorentzian form [40,41] solving the Fresnel equations for the dielectric–metal interface and the expression has been proven valid also for grating [21]:

(3) $$R=1-\frac{4\Gamma_{i}\Gamma_{r}}{\Delta k_{t}^{2}+{\left(\Gamma_{i}+\Gamma_{r}\right)}^{2}},$$

where $\Delta k_{t}$ is the momentum mismatch between the SPP and the diffracted light momentum, null at resonance, while $\Gamma_{i}$ and $\Gamma_{r}$ are the intrinsic and radiative loss, respectively. The first only depends on the intrinsic dissipation of the metal, while the latter depends on the coupling efficiency of the grating [42,43]. Equation (3) is null when the radiative loss equals the intrinsic loss ($\Gamma_{i}=\Gamma_{r}$), corresponding to the optimal plasmonic coupling efficiency, when the energy stored in the SPP mode is maximized. However, it was shown that the derivative of the reflectance over the refraction index is maximized for $\Gamma_{r}=1/2\Gamma_{r}$ [41], therefore the maximum sensitivity is not expected at the best plasmonic efficiency in terms of ∂R/∂n. In our phase interrogation system, we sought to optimize the phase shift at a given refraction index, whose dependence over *R* is not analytically known. Thus, to study phase contribution in different conditions, the use of numerical simulations is fundamental.

**Figure 2:**
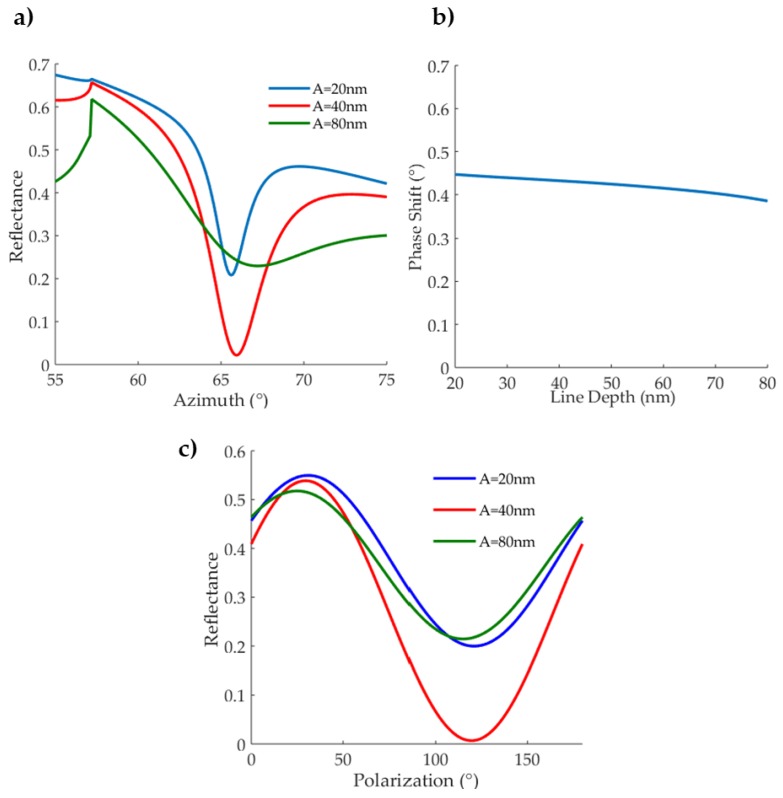
Grating line depth numerical analysis. (**a**) Azimuthal spectrum for 400 nm gratings at 633 nm wavelength at 60° incidence, for different grating line depths: A = 20 nm, A = 40 nm and A = 80 nm (see figure colour legend). (**b**) Phase shift dependence on grating line depth at 60° polar angle, 633 nm wavelength at the corresponding resonance azimuth, for a refraction index of 0.002 from pure water. (**c**) Simulated polarization spectra in water for different grating line depth at resonance. The minimum reflectance is achieved with gratings of 40 nm depth.

The effect of the line depth was studied trough C-method simulations. The line depth affects the radiative loss, determining a change in the plasmonic efficiency. As predicted by the Lorentzian dependence (Equation (3)), it controls the full width half maximum (FWHM) of the plasmonic dip, along with the minimum reflectance at resonance. This can be seen in Figure 2a, where the azimuthal spectrum is reported at a fixed polar angle for different grating line depths. It is apparent that also the resonant azimuth has a slight dependence over the line depth. While the sensing measurements are carried out by changing the polarisation, azimuthal spectra are still relevant, because they represent what is done experimentally to find the resonant azimuth for a given polar angle. For high line depth, hence higher radiative losses, the azimuthal spectrum is broadened, causing low variations of the reflectance around resonance. This makes it harder to find the precise resonance position, which is taken as a reference angle.

Because of the dependence of the resonant angles with the line depth, the phase shift was simulated at a fixed polar angle (60°) but calculating the resonant azimuth for each value of A. The refraction index shift was set at 0.002 RIU (Refraction Index Unit) from water. In that way, it can be seen that the sensitivity varies slightly at resonance with the line depth of the grating (Figure 2b). Smaller depths than the maximum plasmonic efficiency (A=40 nm) were found to increase the phase-shift. However, the sensitivity enhancement comes with a reduction of the polarization spectrum amplitudes (Figure 2c). In detail, the sensitivity enhancement at A = 20 nm is only 3.5% in respect to 40 nm, while the amplitude of the sinusoid in the polarisation spectrum is reduced of 50% (Figure 2b,c). That is why we considered a 40 nm depth most suitable for biosensing experiments as a compromise between phase shift and signal to noise ratio.

### 3.3. Polar Angles Optimization for Sensitivity Enhancement

Our SPR system performance depends on the fabrication parameters of the grating but also on the working conditions of the sensing prototype. The effect of the choice of the polar angle on the phase response was analysed and tested experimentally. The simulations were performed by C-method, considering a grating of 400 nm period and 40 nm line depth in water, with a wavelength of 633 nm, always calculating the resonant azimuth for the given polar angle. The polar angle is relative to the air interface, so the real incidence angle is lowered according to Snell’s law.

**Figure 3:**
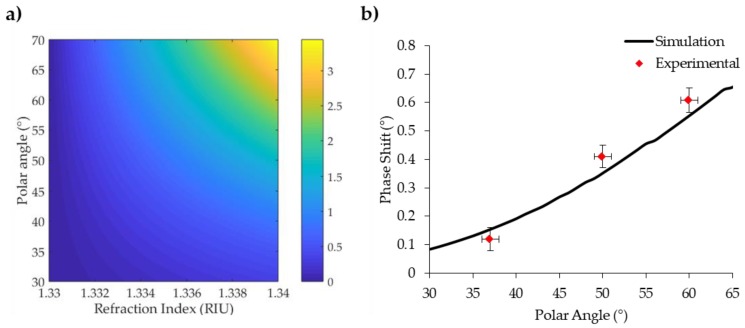
Numerical simulations and experimental results of polar contribution to phase shift enhancement. (**a**) Phase shift colour map of gold gratings with a 400 nm period in water, varying the polar angle in resonance condition. Incident wavelength: 633 nm. (**b**) Experimental phase shift enhancement with polar incidence angle. Red dots represent experimental data; the black line is the simulated value for the corresponding refraction index shift extrapolated from the colour map. s.e.m. of the points are shown.

In Figure 3a the phase shift for different polar angles is reported as a colour map varying the refractive index. Moving towards grazing angles allows us to obtain larger phase shifts, but technical constraints of the detection prototype did not allow us to explore angles over 60° from the normal.

Numerical predictions were tested experimentally measuring the SPR response to bulk variations of refraction index from Milli-Q water to a 200 mM solution of NaCl in Milli-Q water. This is a standard procedure adopted to monitor SPR response, and we used a buffer similar to refraction index shift to that expected in sensing application to biomedical research. We compared three different polar angles (37°, 50° and 60°) and experimental results were overlapped to simulations. As shown in Figure 3b, experimental results are in good accordance with simulations. The modification of the polar angle at 60° allowed us to obtain a system with an SPR response 30% larger than previous reports [39].

### 3.4. Azimuthal Angles Optimization for Sensitivity Enhancement

**Figure 4:**
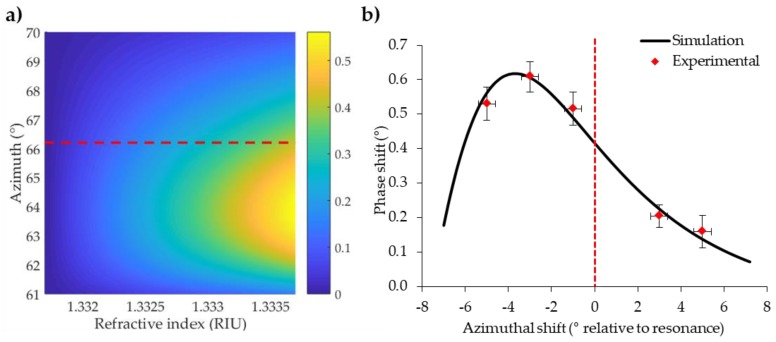
Numerical simulations and experimental results of azimuthal contribution to phase shift enhancement. (**a**) Simulated phase variation colour map of a grating with a period of 400 nm and 633 nm incident wavelength. The polar angle was fixed at 60°, while the azimuth was varied around resonance. The phase variation was simulated for a set of refractive indexes starting from the water. The dotted red line represents the resonant azimuth for water. (**b**) Experimental phase shift for a 200 mM NaCl solution from water (considered as reference), overlapped to the simulated longitudinal section profile from the colour map for different azimuths around resonance, at 60° polar angle. The dotted red line represents the resonant azimuth for water.

After polar incidence optimization, we focused our attention on azimuthal rotation. Until now, the azimuthal orientation was selected based on resonance angle. To this aim, we used numerical simulations previously described to evaluate SPR response enhancement varying azimuthal orientation around the resonance angle. The effect of the azimuth was studied by C-method simulations at a fixed polar angle by varying the azimuth around resonance (Figure 4a). The monitored parameter is the fitted phase shift between $n_{0}$ = 1.330 and $n_{1}$ = 1.332. The results showed that the larger SPR response is found before resonance. Thus, the resonance angle is not the best condition, in particular for low refractive index changes. This is not surprising, since when monitoring the reflected intensity also for prism-coupled devices the ∂R/∂n is not costant but has a maximum for a specific refraction index variation [21], due to the non-linearity of the R(n) function, which is also true for gratings.

This observation was then confirmed experimentally by measuring SPR response to bulk refraction index change given by 200 mM NaCl from Milli-Q water, as is done for polar angle optimization. A few-degree rotation scan of the azimuth angle around resonance was performed and overlapping simulation results with experimental data showed good agreement (Figure 4b). Careful azimuthal orientation around resonance is particularly relevant in an aqueous environment, increasing by up to 30% the phase response for this refraction index variation.

This angular shift of 0.6° in polarization corresponds to 0.002 RIU thus resulting in a system sensitivity of around 300°/RIU, a significant increment with respect to previous reports from our laboratory that gave results lower than 200°/RIU for similar saline solution measurements [30]. This demonstrates that accurate optimization of experimental parameters is capable of providing a significant increase in sensing response.

### 3.5. GC-SPR Response Enhancement: Biotin-PEG-Thiol Functionalization and Biorecognition with Avidin

**Figure 5:**
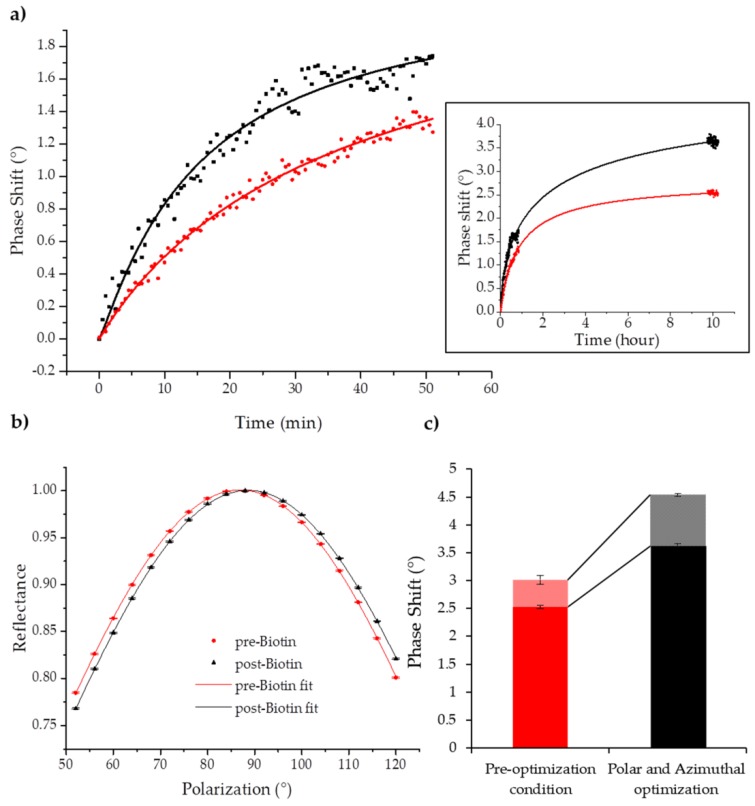
GC-SPR biosensing enhancement upon polar and azimuthal angles optimization. Biotin-PEG-SH functionalization shows higher phase shift in respect to “pre-optimization” configuration (in red) for “post-optimization” configuration (in black) in the first hour of the experiment (**a**) and after several hours when the functionalization is almost complete (inset). Data (square dots) were fitted with Hill curves (lines) obtaining values of the reduced *R*^2^ > 0.999. (**b**) Representative curves of the polar spectrum at t = 0 and t = 10 h of Biotin functionalization. (**c**) Quantification of complete experimental results in terms of phase shift for the non-optimized (in red) and optimized (in black) configurations. Full colour bars quantify the phase shift due to Biotin-PEG-SH functionalization signal, while patterned bars quantify the subsequent Avidin binding to Biotin. Data are reported with s.e.m.

Since this GC-SPR sensitivity enhancement study was devoted to improving biorecognition experimental results, the biotin–avidin reaction was used as a reference method to analyse SPR performance in a biosensing scenario. A 2 mM Biotin-PEG-thiol 2 kDa solution was fluxed into the microfluidic cell as described in Section 2.2. Biotin functionalization was monitored and compared in the “pre-optimization” (in red) and “post-optimization” (in black) conditions and experimental data (square dots) and corresponding fitting curves (lines) are shown in Figure 5a.

The functionalization kinetics was monitored to see the avidin–biotin binding. Larger responses upon optimization were clearly visible even in the first hour of the experiment. Upon saturation with biotin, we obtained a 40% increase in SPR response, which was in line with the salt solution calibration previously shown. For completeness, two representative polarization spectra are shown in Figure 5b at t = 0 and t = 10 h of the Biotin functionalization protocol, from which $\Delta \alpha_{0}$ can be extrapolated by fitting the experimental data. Moreover, the phase shift caused by Avidin binding was significantly larger, almost doubled (Figure 5c).

## 4. Discussion

In this study, we took advantage of numerical methods to enhance the performance of a custom-made on-bench system based on phase-interrogation for SPR sensing. From numerical simulations, it was possible to optimize different parameters regarding both the grating fabrication procedure and the sensing prototype. However, translation from numerical simulations to experiments imposes some practical constraints: all parameters should be carefully selected to ensure response enhancement without compromising signal-to-noise ratio or introducing beam distortions. In GC-SPR, the correspondence between grating parameters and incident laser wavelength is crucial. The line depth of the grating is a parameter that influences the plasmonic efficiency and sensitivity, but the two cannot be optimized simultaneously [41]. In the range of line depths considered, the sensitivity does not change dramatically, making the method robust and tolerant to mild variations on the fabrication procedure. The 40 nm depth, although predicted not to have the best sensitivity, ensures an easier identification of the resonant azimuthal angle as well as maximizing the signal-to-noise ratio of reflectance in polarization scan.

The major technical constraint imposed by mechanical parts of the set-up concerned polar incidence. Although numerical simulations predict that higher angles than 60° would ensure a further increase in sensitivity, a further grazing incidence was tested but, in addition to mechanical limitations, it induced an enlargement of the laser spot, introducing unwanted aberrations, compromising SPR detection. Eventually, the proper azimuthal orientation of the grating led us to an increase from 30% to 50% in sensitivity. The optimal azimuthal orientation is not constant for all refraction index variations since the response is not linear. Thus, upon resonance identification, the most efficient orientation can be predicted by numerical simulations and adjusted prior to the experiment.

The novel optimization of the system results in a sensitivity up to 300°/RIU, that is up to 50% larger than previously obtained with the same apparatus [30]. Comparison with other systems based on plasmonic structures is complicated by the variety of sensing methods adopted by different group, but can be possible through the resolution expressed in terms of RIU. For all systems, this parameter results from: Resolution=Measurement Error/Sensitivity, where the error is calculated over at least 10 repeated measurements at steady state. System resolution depends on intrinsic (sensing area) and extrinsic (signal acquisition, apparatus optics and mechanics, temperature control) factors. Our system has a resolution of 3 × 10^−5^ RIU in line with other reports using plasmonic crystals [44,45], but it can be further increased acting mainly on extrinsic parameters, such as temperature control and angular accuracy of the servomotor adopted, which was found to be a major source of instability in repeated measurements. The need for a more sensitive system, while maintaining the simplicity and cost-effectiveness of the apparatus, is crucial for applications to clinically relevant biochemical interaction studies. The platform here presented lays the bases for future investigations involving molecular and biophysics phenomena more complex than avidin–biotin recognition. Among others, the system is currently challenged for leukaemia cell counting and label-free leukaemia cell-drug interaction analysis [46], this latter being a field of application poorly explored with GC-SPR. This application can open new possibilities toward personalized medicine in all those clinical conditions where the number of available cells from patients is scarce and a rapid, real-time monitoring is desirable, as paediatric leukaemia. Moreover, a major advantage of gratings lays on the versatility and miniaturization capabilities of the configurations that can be fabricated. Among others, the plasmonic chip can be integrated into a system with surface acoustic waves (SAWs) to promote analyte mixing and binding [31]. Furthermore, a chip with multiple sensing areas can be exploited for multiplexed analysis without expensive microspotter systems to evaluate different analytes simultaneously [47].

## 5. Conclusions

The limitations on the exploitation of GC-SPR systems are mainly attributable to the lower sensitivity of the apparatus compared to prism-coupled systems. On the other hand, the use of gratings enables extreme miniaturization and integration of the sensing areas within lab-on-a-chip systems. Here, we investigated a GC-SPR detection setup based on phase interrogation with the biosensing area integrated in a microfluidic chamber, which has been successfully optimized to enhance global response to bulk refraction index variations and protein adhesion and recognition. The most suitable working parameters to increase the SPR response were studied with a simulation and then tested experimentally. This brought an observed increase of 30–50% in the global response, which makes the sensing performances of gratings couplers more competitive and appealing. Further optimization may be obtained by, for example, equipping the sensing instrumentation with a more accurate servomotor.

The SPR response enhancement in the aqueous medium reported in this work is capable of promoting the integration of GC-SPR devices for novel applications in biosensing in more complex lab-on-a-chip platforms.
